# Exploring Attitudes toward Animal Welfare through the Lens of Subjectivity—An Application of Q-Methodology

**DOI:** 10.3390/ani10081364

**Published:** 2020-08-06

**Authors:** Yari Vecchio, Gregorio Pauselli, Felice Adinolfi

**Affiliations:** Department of Veterinary Medical Sciences, University of Bologna, 40064 Ozzano dell’Emilia (Bo), Italy; yari.vecchio@unibo.it (Y.V.); felice.adinolfi@unibo.it (F.A.)

**Keywords:** animal welfare, opinions, Q-methodology, policymaking, discourses

## Abstract

**Simple Summary:**

In this paper, we offer a methodological solution to the policymaker to improve the structure of the surveys used for public consultations. Over the years, we noticed the importance of opinions in legislative processes, particularly in animal welfare. The issue, to which European citizens have historically been sensitive, has been the target of multiple consultations aimed at considering perceptions about farming conditions. However, standard surveys suffer from bias and design errors. To overcome these problems, we propose the use of Q-methodology to understand the opinions of veterinary students. The results contribute to the improvement of traditional surveys used to understand what people think about animal welfare and could be useful in providing information to the policy making process.

**Abstract:**

Opinions increasingly influence legislative processes. The case of animal welfare (AW) standards is a clear example of the role played by opinions in political decisions. The issue, to which European citizens have historically been sensitive, has been the subject of numerous consultations and investigations, aimed at considering citizens’ opinions regarding breeding conditions. However, these tools and in particular standard surveys, suffer from response biases such as the prejudices involved in the design of questions and the interpretation of the results. To mitigate these problems, we used the Q-methodology, which is an inductive but systematic methodology focused on patterns that explain the ideas of individuals. The purposive sample consisted of 36 veterinary students who were acquiring scientific knowledge about AW. The results, in addition to providing policymakers with food for thought for the further development of AW standards, contribute to testing the use of alternative methodologies to collect citizens’ views. This mapping of viewpoints helps to build a more effective form of AW policy making.

## 1. Introduction

European legislation on animal welfare (AW) is rooted in a long tradition that has seen some European Union (EU) countries act as pioneers in setting minimum standards [1]. The start of European and international AW legislation was the first law against cruelty inflicted on animals, the Martin’s Act, adopted in England in 1822 [2]. It was succeeded in the first half of the 20th century by the adoption of ad hoc legislative packages in England, Denmark, Germany, Norway and Sweden [1,2]. The cornerstone of EU legislation in this domain was the Directive on the stunning of animals before slaughter, adopted in 1974 [3]. It was followed by the Directive on the protection of animals during international transport in 1977 [4]; since 1999, the public interest in AW has been embedded in the European Constitution. Because of to a specific protocol, annexed to the Treaty of Amsterdam [5,6] the concept of animal welfare impact assessment was introduced, implying EU policies had to fully consider AW implications [1]. The current EU legislation on AW has resulted in an extensive body of norms regulating minimum standards in livestock production, covering all sectors and stages of production from farm to slaughter [7,8]. The EU defines the limits within which livestock farmers are considered socially acceptable by setting direct legal standards for AW—below these, farmers’ practices are considered detrimental to public interest [9,10,11]; above these, reconciling supply and demand for AW is the responsibility of market regulation [12,13,14,15,16]. The 2006–2010 EU Action Plan [17] and the 2012–2015 EU AW Strategy [18] stress both AW as a public good, opening up to the extension of minimum legal standards and AW as marketable good, recognizing how information asymmetries have become a matter of public interest [19,20].

The long history of European legislation on AW has witnessed the growing contribution of opinions in building AW policies [21] and few discourses between Brussels and Strasbourg on the subject fail to mention data from public consultations. The growing power of citizens has pushed many scholars to focus on a more holistic knowledge about their attitudes [22,23,24], promoting the integration of some forms of subjectivism and related epistemology into the analyses [25,26,27]. This has favored the development of alternatives to traditional positivist approaches and these mixed methods have gained importance in recent decades [28]. The combination of qualitative and quantitative techniques has shown a valuable adaptability to the practical purposes of researchers [29]. In particular, the Q-methodology (QM) has become popular in the study of perceptions and attitudes toward ethical attributes [30], enabling the extraction of individual opinion and constructs into a formal methodological design. This method provides new insights that may be useful to build better generalized surveys, especially in a context deeply influenced by the bias effects derived from the political and social backgrounds.

The research question that inspired the work is how opinions are interpreted and codified in policy processes, assuming that (1) standard surveys suffer from bias that do not allow the interpretation of perception and (2) the use of hybrid methods could improve the interpretation of viewpoints to be used in the subsequent creation of generalized surveys.

We applied the QM to a sample of veterinary students who have chosen to specialize in animal production. The study of animal welfare is a substantial part of veterinary education [31] and the identified group of students had already established an advanced level of knowledge on the subject. In the coming years, most of the students will probably be involved in the livestock sector and in animal public health, influencing the meaning of animal welfare in these areas. After an examination of the role of opinions in influencing AW policies and a review of the role of attitudes in human decision-making process, we used the QM results to offer further inputs to the dialogue between institutions and citizens, which could provide suggestions to the policymakers to build better generalized surveys.

## 2. The Role of Opinions in Influencing the AW Legislative Process

Opinions already played a decisive role when the Martin Act was passed. The first legislation that made cruelly beating, abusing or ill-treating certain animals punishable by fines and imprisonment was the result of the reaction of English citizens to the conditions of animals used massively on the streets [32]. Two years later, the Society for the Prevention of Cruelty to Animals was founded and in 1840, Queen Victoria became the patron, changing the name of the society in Royal Society for the Prevention of Cruelty to Animals (RSPCA). The number and voice of AW associations rose rapidly and when the high numbers of dead horses during the Anglo–Boer War (1899–1902) became public [33], the RSPCA referred to war horses as “war workers” or veterans, deserving compassion as much as any human worker or soldier [34]. Many AW organizations began to monitor the treatment of war horses by putting pressure on military forces to improve AW [35]. During this period, public sentiment toward AW gained ground in many countries; the first national legislation preventing cruelty and unnecessary suffering was developed in England in 1911, followed by Denmark in 1916, Germany in 1933, Norway in 1935 and Sweden in 1944 [2].

After the Second World War, attention shifted to the conditions of farmed animals. The publication of Ruth Harrison’s book Animal Machines in 1964 can be considered a milestone in the critique of modern farming methods. It led the English Parliament to publish the Brambell’s Report [36], which provided a definition of AW and has become the beacon of modern AW policies [37,38]. A few years later, in 1974, the European Economic Community (EEC) approved the first common AW legislation, the Council Directive 74/577/EEC, on the stunning of animals before slaughter [3]. In the second half of the 1980s, Bovine Spongiform Encephalopathy (BSE) catalyzed public attention on the living conditions of animals [39] and raised many concerns about human health [9,40]. Since then, AW has occupied an important space in the media and the active role of citizens and associations has grown significantly. This has resulted in a dual response from European policymakers—the raising of the minimum standards for the protection of all farmed animals [41] and the strengthening of the legal basis for AW in the European Community (EC) treaties [2,42] and the promotion of a continuous dialogue with citizens and stakeholders. The EU now works systematically on AW on the basis of scientific evidence but also under the pressure of its citizens [43]. The latter has been given a crucial role in the development of European legislation [44,45] and investigations of opinions have become of major importance in setting the EU agenda on AW [17]. The Community Action Plan on the Protection and Welfare of Animals 2006–2010 [17], which led to the introduction of animal-based indicators in EU legislation (Directive 2007/43/EC [46] and Regulation No 1099/2009 [47]), was the subject of a wide public consultation; the European Union Strategy for the Protection and Welfare of Animals 2012–2015 [18] took data from Eurobarometer surveys as its main reference point, in particular to highlight the market failure of private standards. Although one in seven European citizens consider animal welfare as a priority [48], the market for private standards is still poorly developed [49] and the potential of these products is unused [50]. More than in other policy areas, the tool of citizen surveys provides key data of the narrative underlying the political work produced by the European institutions on this subject [50,51]. The European Court of Auditors’ Report on AW of November 2018 [52] and the Report of the Online Consultation on the Future of Europe of April 2019 [48] are only some examples of how the policy-making process considers the opinions expressed by citizens on the AW issue. Therefore, the outcome of the political process, although mediated by the interests of other actors and the institutional framework [53], tends to be strongly linked to the emerging interpretation of the social acceptability of AW policies [45] and consequently the pressure of voters [44]. More generally, the theory and, especially, the practice of public administration are increasingly oriented to look at the citizen not only as a target of policies but also as an agent in their design [54,55,56]. Since the late 1970s, political decision-making processes have seen citizens’ power grow rapidly [57].

The consequence has been the development of various procedures allowing the direct participation of citizens in the creation of public policies. In Europe, the presence of forms of direct citizen participation has become an essential element of political legitimacy, which is recognized in Article 11 of Treaty on European Union (TEU). The functioning of this principle is often guaranteed through the open consultation mechanisms activated by the Commission to gather the views of citizens and stakeholders [58]. The EU has implemented a system of periodic surveys to collect information on the attitudes of its citizens toward major European issues and, for over 40 years, the Eurobarometer has been providing data on their opinions and monitoring the evolution [59]. The data collected by Eurobarometer, as well as the results of public consultations, influence the legislative process [60,61,62] as they become an essential part of the process. The EU has developed a series of initiatives promoting dialogue with citizens with the aim of giving due consideration to interdependencies with different values, norms and interests [63], especially in cases such as AW, where the production of knowledge is conditioned by the political context and the society in which knowledge production spaces are embedded [63,64,65,66]. The impossibility of separating knowledge and the knower has led to the progressive acceptance of the fact that subjective meanings differ from objective meanings, influencing the perception of problems and consequent policy choices. The involvement of citizens promotes the integration of collective values and preferences in policy design [67,68,69,70]. This requires focusing on subjective points of view and the nature of this effort is difficult to reconcile with quantitative approaches [71,72,73]. Although analytical approaches are unlikely to capture the different facets of human subjectivity, the results of qualitative analysis could be ambiguous and difficult to generalize [74].

## 3. Materials and Methods

Growing amounts of literature identify the use of hybrid methodologies as a possible solution [75,76,77]; among these, the QM is increasingly appreciated by researchers and analysts [30,78,79,80]. It is an inductive methodology [81] that provides a systematic and quantitative elicitation of respondents’ values and positions. Formalized by Stephenson in 1935 [82], the QM is a qualitative and quantitative approach used to define the discourses that frame people’s points of view on certain topics, particularly useful in the analysis of complex and socially controversial issues [83,84]. Using factorial analysis, the QM investigates similarities and differences in respondents’ points of view [85,86] and assumes them as common points of view by capturing significant groups of correlations. Unlike the R methodology, which involves correlation and factorization of traits in a sample of individuals, in the QM, individuals are correlated through the classification of discourses [87]. QM focuses on the degree of similarity between answers in line with its subjective nature, whereas the R method investigates the relationship between objective variables following its objective essence [88]. Therefore, the results of the QM are one or more points of views, whereas the results of the R methodology analytically describe the characteristics of a population statistically associated with different opinions or attitudes [89]. The QM could be defined as an approach applied to the study of human subjectivity [90], which allows exploring the system of values and motivations that guide people’s behavior [89,90].

The QM was first applied to marketing, referring to the clustering attempts of Sommers [91,92], Stephenson [93,94,95], Schlinger [96] and Martin and Reynolds [97], up to the study of Smith and Albaum [98], who demonstrated how this technique is useful for identifying the corporate image. It has recently been proposed as a valid alternative that could overcome the limits of the external perspective in the market demographic segmentation [99,100]. Furthermore, interest is growing in studies using the Q-methodology for topics such as political science [101], environmental issues [86,90] and food consumption choices. It has already been applied to the study of food security [102], the promotion of healthy food environments [81,103], the investigation of food trust [30], the role of the information contained in the certifications in influencing the purchasing choices of food products [30] and to the attitudes toward AW [80].

Over time, the QM has undergone variations in the application of the procedural steps; it has been proposed with 4 [104], 5 [101,105] and 6 steps [86,106,107]. During the years, the 5-step procedure has been the most used and has become the standard version after the work of McKeown and Thomas [90]. The procedure we used involved—construction of the concourse, development of the Q set, selection of the P set, Q sorting and Q factor analysis and interpretation.

In the first step, which aims to identify the concourse, it is necessary to research all the points of view on the problem under consideration. The concourse involves the communication of the subjectivity of all possible aspects that can be related to a given topic [108,109]. At this step, all the aspects that the subject has investigated must be considered. A necessary condition for considering all aspects is the use of a focus group composed of researchers that are familiar with the topic [82,108,110]. The focus group was composed of six researchers, four of whom represented experts in pig, cattle, poultry, sheep and goat farming, with the other two being experts in marketing and survey methodologies. The ideas were also collected through secondary sources such as literature [110,111]. In the end, over 70 statements useful for the investigation were identified. In our case, too many statements were identified and we compressed them to obtain a better result and facilitate the following step [112].

In the second step (Q-set), we selected the functional statements for the investigation from the previous list [101,113]. This operation is the heart of the analysis, since the choice of statements can condition the success of the analysis [101]. These should constitute a variety of different opinions and should not necessarily represent concrete facts but also sensations [105]. There were 41 sentences left after eliminating all those that could mislead the study or that could be factual and therefore not express any opinion [112]. These were printed on small sheets of paper, identifiable with a code useful for the recognition [105] that could not influence the participant in any way. Finally, as suggested by Brown [101], the test was validated by a colleague before being administered to the sample.

During the third step, consisting of the selection of the participants (P-set), we constructed a purposive sample [103,114] of 36 veterinary students who had completed the first three years of studies and were attending the first year of a master’s degree in animal production. This was justified by their strong knowledge of animal welfare and their familiarity with the various facets of animal husbandry [115,116,117,118,119,120]. They have the potential to influence the meaning of animal welfare in the future by becoming stakeholders or being involved in policy processes. This is also supported by the indications of Watts and Stenner [89], who suggest that the respondents should be at least half of the sample and by following Brown [101] and Van Exel and Graaf [121], who claimed that the group should be composed of people who are theoretically relevant to the research question.

**Figure 1:**
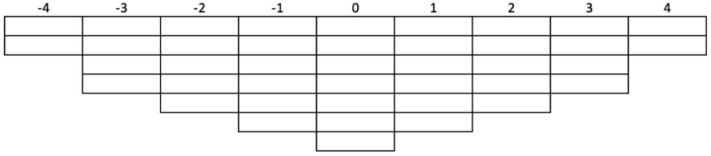
Fixed quasi-normal distribution. Ranking values range from −4, through 0, to +4. A total of 41 items can be accommodated in the distribution illustrated.

The fourth step consists of Q-sorting, where each participant puts the statements in order [122]. It occurs in two phases, both with written instructions. Participants initially classify the declarations in three groups—agreement, disagreement, or neutrality [110]. After this phase, the same statements must be placed in a distribution grid with a number of spaces corresponding to the total of statements. In our case, the grid (Figure 1) was built with limits ranging from “completely disagree” (−4) through “neutral” (0) to “completely agree” (+4) [101]. These spaces and those existing between them (−3, −2, −1, +1, +2, +3) force the participants to classify the statements on AW according to the intensity of their disagreement, neutrality or agreement. We used a forced symmetric distribution [117] that obliges respondents to catalog the statements in the predefined grid [112].

The last step is performing an inverted factor analysis [83,123,124,125], since a typical factor analysis focuses on the discovery of correlations between variables, whereas the Q-methodology aims to show the correlations between people [89]. This type of approach can be considered as the key element of quantitative application [112]. KADE software [126] was used to analyze the data.

## 4. Results

An intercorrelation matrix was constructed, which was factor-analyzed using the centroid procedure [86] and the solution was rotated using the varimax criteria [85]. The next step was to determine the criteria for the choice of factors. First, only eight factors with a value higher than one were chosen [127]; of these, only four were analyzed based on the interpretability criteria [85]. Only those defined by at least three individuals and identifiable with distinctive characteristics were selected. The characteristics of the factors are represented in Table 1.

We discovered four ideal type Q sorts, as shown in Table 2.

The factor scores are weighted averages (Z-scores) of the values given at each instruction by the individuals defining the factor. In Table 2, the Z-scores were converted to the values of the original scale [−4 to +4] to facilitate the interpretation of the result [112]. The table shows us the value that each statement has in each of the four groups that emerged from the factorial analysis and its reading of the table immediately displays the differences and similarities between the different points of view.

### 4.1. Discourse A: Idealists

This discourse was composed of nine students involved in the survey who shared a strong concern for AW. This concern is reflected in individual behavior, through the choice of products that meet higher welfare standards and in collective responsibility, with the European legislator being asked to ensure higher mandatory standards. We identified a strong belief in the connection between AW and the healthiness of products. This attention to welfare is accompanied by interest in environmental issues in a vision that connects AW, ecology and the welfare of citizens.

### 4.2. Discourse B: Food-Addicted Beyond AW

The second extracted point of view was composed of three individuals who shared little interest in the AW attribute. Indifference was demonstrated by individuals buying products based on convenience rather than welfare. This behavior is probably conditioned by their low interest in decreasing meat consumption and above all by not recognizing the ethical value of the attribute. Despite this consideration, the discourse included individuals who shared the desire to share experiences; once they have tried a product and found it good and satisfactory, they tend to suggest it to others, especially friends and family. They also agree that food, cooking and the choice of places to eat out are among the most frequently discussed topics.

### 4.3. Discourse C: Environmentalists

The idea shared by the 13 respondents of this group was the importance of the environment and that livestock farms are crucial for sustainability. In this group, as in the first, there is a strong relationship between human health and meat consumption. These people firmly believe that public action is crucial for the improvement of AW standards. The sensitivity of this group is reflected in their purchasing choices that reward the high quality of welfare standards, whereas the least important thing is the reputation of the brands.

### 4.4. Discourse D: Pragmatic

These three individuals shared a strong interest in AW. They fully agreed that current European legislation guarantees high standards of welfare and that it coincides with the physical and mental welfare of the animal at all stages. They also believe that substances that can harm livestock health affect consumers who eat their products but strongly disagree with the administration of antibiotics through feed being unnatural. The interest in AW was also reflected in the common belief that they want to protect the environment and avoid depletion of natural resources caused by the livestock sector.

Given these results emerged, a correlation analysis was conducted among the extracted discourses to look for any significant correlations that would allow a better interpretation of the interviewees’ points of view. Table 3 shows that the correlation analysis identified a strong relationship (0.64) between Discourses A and C. This strengthens the emerging link between AW and environmentalism. The correlation between these two points of view explains how the perspectives of these ideas are linked to each other and find different points of contact. This is in contrast to other points of view defined as “Pragmatic” and “Food-Addicted Beyond AW,” which are more linked to the sphere of daily routine. The four points of view have an even stronger value since they come from a programmed sample, which was not influenced by false information since they know the theme of animal welfare because of their studies.

## 5. Discussion and Conclusions

The analysis allowed us to extract relevant discourses and to compare between groups. From this, some common aspects and some differences emerged that help to understand people’s attitudes. The groups showed a positive trend toward environmental issues and claimed that the purchase of sustainable products can increase the welfare of consumers and citizens. At the time of buying these products, no individual declared to be influenced by the media. People in the four discourses believed that substances administered to animals, such as antibiotics, could affect consumer health, in accordance with the One Health principle in which human, animal and environmental health are integrated [128]. The link between the quality of the feed and the quality of the final product is commonly recognized but AW is not necessarily connected to a better product taste. AW is identified by all as a condition of physical and mental health to be achieved at every stage of the process. A significant correlation emerged between the discourse of the “Idealists” and the “Environmentalists,” strengthening the connection between environmentalism and interest in AW. These two discourses paid attention to the attribute of AW at the moment of the purchase, refusing to give up this feature in exchange for “a good product” and stated that labelling schemes should clearly indicate the level of AW standards. Both Idealists and Environmentalists responded that the administration of antibiotics in feed is unnatural and that AW legislation should meet the highest standards. In a different way, the Food-Addicted Beyond AW and the Pragmatists were in favor of antibiotics in feed and did not require the highest level of welfare from the legislation. The Pragmatists also believed that the current European legislation already guarantees high standards of AW, whereas the Idealists strongly disagreed with this concept. The greater sensitivity of the Idealists to these issues was confirmed by being the only ones who considered reducing or eliminating meat from their diet after watching documentaries on intensive farms. The individuals of the Food-Addicted Beyond AW group were the only ones who did not pay attention to welfare when faced with a convenient product and were the least interested in protecting the environment from the damage caused by farming. The individuals of this group confirmed their particular attitude by suggesting to acquaintances the purchases that have satisfied them and recognizing the importance of distinguishing themselves from the others.

The results obtained from interviews with veterinary students allowed us to highlight the similarities and differences between different points of view [89] and to explore the motivations of people [89,90,129,130]. The QM has been proved to be a useful tool for interpreting these answers [82] even if it poses limits derived from the exploratory nature of the methodology and from the use of a purposive sample [131]. Although many authors boldly generalize the results of this type of survey obtained with random samples [86,132,133], we are aware of this limitation and have considered it, especially when choosing such a specific sample. The choice of interviewing veterinary students, conscious of the topic, allowed us to go beyond the results that would be obtained from descriptive statistics. The indications that emerged from this work can therefore be useful for two reasons—for the particular composition of the sample and for the methodology used in the exploration of consumer attitudes.

The purpose of this work was to provide policymakers with new methodological insights that may be useful for enriching the creation of traditional surveys, such as those of the Eurobarometer (e.g., Reference [134]), which analyze issues influenced by ethical attributes like AW. Institutions work both on scientific evidence and public pressure [35], especially in Europe [35,53], where citizens’ opinions have been incorporated in several documents concerning AW over the years. Therefore, it is important to explore the opinion of citizens by eliminating the bias effects derives from the political and social contexts [56]. The collective values of citizens, which have become part of the legislative process and have to be considered [70,135], are hardly captured by the purely quantitative or qualitative approaches used by standard surveys [75]. In this study, we overcame these limits using a hybrid methodology that allowed us to explore the subjectivity of respondents with respect to an issue influenced by the social context, such as AW [85]. Our findings contribute to the discussion on the perception of animal welfare with a methodology to support standard investigations during the design phase. Due to its exploratory nature, the study did not aim to provide an alternative methodology but rather offers a methodological improvement that allows a better understanding of the points of view of purposive samples. These are composed of people who are familiar with the topic, so that the results can then be used for the construction of generalized surveys for citizens. The four points of view that emerged from our analysis therefore do not represent the public opinion of citizens but represent four idealizing factors that were extracted from a group of people with strong knowledge of the subject. These aspects should represent starting points to be investigated during public consultations to understand if there are relationships between idealists and people who care about the environment or, vice versa, how to relate to those for whom the issue of animal welfare is not a priority.

## Figures and Tables

**Table 1:** Factor characteristics.

Factor	Factor 1	Factor 2	Factor 3	Factor 4
No. of Defining Variables	9	3	13	3
Composite Reliability	0.973	0.923	0.981	0.923
Standard Error of Factor Z-scores	0.164	0.277	0.138	0.277

**Table 2:** Statements with scores on each of the five extracted discourses.

No.	Statement	A	B	C	D
1	Substances that may harm animal health affect the health of consumers who eat their meat and/or meat products.	4	1	4	3
2	Animal Welfare legislation must ensure compliance with maximum AW standards.	4	0	3	0
3	I know what AW is but I do not pay any attention to this feature when I purchase.	−3	0	−1	0
4	I often discuss the subject of AW with friends and colleagues.	1	−2	0	−1
5	If a product is good, I buy it regardless of AW.	−3	3	−1	2
6	Current European legislation ensures high standards of AW.	−4	0	1	4
7	It is important to protect the environment and avoid the depletion of natural resources in the production of livestock products.	3	1	4	3
8	The administration of antibiotics in animal feed is unnatural.	2	−3	2	−4
9	What the animals eat is crucial for their well-being.	1	1	3	2
10	I would buy products that meet high AW standards at a higher price if it was clear what contribution I made to the community through the standards I adopted.	1	−1	−1	2
11	Italian livestock production meets the highest AW standards in the world.	−1	−1	0	1
12	I really like shopping.	−1	−2	−4	3
13	The reputation of the brands is fundamental in my food purchasing choices.	−1	−1	−3	0
14	Once I have tried a product and found it good and satisfying, I tend to suggest it to others, especially friends and family.	0	4	1	0
15	I am very interested in environmental issues.	3	2	2	3
16	No one in my family or friends has ever suggested that I pay attention to AW.	−4	−1	−1	1
17	I care a lot about the quality of my food.	0	2	0	1
18	I am very interested in social issues, particularly ethical issues.	2	0	−1	−1
19	AW is a matter of particular concern to farmers.	−2	−2	0	−2
20	After watching some video documents (documentaries, broadcasts, etc.) on intensive farming, I have seriously considered the possibility of eliminating/reducing significantly the consumption of meat in my diet.	2	−4	−2	−1
21	AW should coincide with the physical and mental well-being of the animal at all stages of the production cycle, including transport and slaughter, if any.	3	1	3	4
22	Products that meet high standards of AW are still too expensive to be permanently included in my diet.	−2	1	1	1
23	I believe that references to AW on products are primarily a marketing activity.	0	0	−2	0
24	Products that meet high AW standards are difficult to find.	0	−3	0	−1
25	Increased consumption of products that meet high AW standards promotes traditional and extensive production methods.	2	−2	1	−2
26	I think eating styles say a lot about the person.	0	2	1	−1
27	If a food product meets my expectations, I tend to buy it even if it is expensive.	1	3	0	−1
28	I prefer products that meet high standards of AW because they taste better.	−1	−3	−3	−3
29	Products that meet high AW standards are safer for human health.	0	−1	1	0
30	AW on farms is essentially guaranteed by sufficient and clean spaces.	−3	0	−2	−2
31	Products that meet high AW standards are difficult to identify.	1	−2	0	1
32	Labelling schemes should clearly indicate the level of AW standards.	2	1	2	0
33	I doubt that buying sustainable or ethical products will increase the welfare of consumers and citizens.	−3	−1	−2	−3
34	Higher AW standards coincide with higher quality standards for meat and meat products.	0	0	3	1
35	Intensive farming systems are unnatural.	3	2	2	−2
36	Cooking and food media influence my purchases.	−2	−4	−3	−4
37	Buying sustainable products makes me feel like I’ve done a good deed.	−1	−3	−3	2
38	Distinguishing myself from others is very important to me.	−2	3	−4	−3
39	If a product is convenient, I buy it regardless of AW.	−2	4	−1	−3
40	The food, the kitchen, the choice of places to eat out are among the topics of discussion that I face most frequently.	−1	3	−2	−2
41	Animal feed is decisive for the final quality of the products.	1	2	2	2

**Table 3:** Factor score correlations.

Discourses	Idealists	Food-Addicted beyond AW	Environmentalists	Pragmatic
Idealists	1	0.0527	0.6409	0.1757
Food-addicted beyond AW		1	0.2058	0.0927
Environmentalists			1	0.354
Pragmatic				1
